# Risk factors for twin pregnancy in women undergoing double cleavage embryo transfer

**DOI:** 10.1186/s12884-022-04606-1

**Published:** 2022-03-29

**Authors:** Panpan Chen, Kai-Lun Hu, Jiani Jin, Ruixue Chen, Qiqi Xu, Wei Zhao, Runju Zhang, Lanfeng Xing, Yimin Zhu, Dan Zhang

**Affiliations:** Key Laboratory of Reproductive Genetics (Ministry of Education) and Department of Reproductive Endocrinology, Women’s Hospital, Zhejiang University School of Medicine, Hangzhou Zhejiang, 310006 People’s Republic of China

**Keywords:** Twin pregnancy, Low birth weight, Preterm birth, Risk factor

## Abstract

**Background:**

Two or more embryo transfers have been used to increase the success rate of live birth in traditional in vitro fertilization (IVF) strategy at the expense of increased risks of multiple pregnancy and adverse perinatal outcomes. The decision regarding the elective single embryo transfer or double embryo transfer remains inconclusive. The aim of this study was to investigate the risk factors for twin pregnancy in IVF.

**Methods:**

Participants who underwent their first fresh IVF cycle where two cleavage stage embryos were transferred in Women’s Hospital of Zhejiang University between January 2010 and December 2018 were included in this retrospective cohort study. The primary outcome was twin delivery. Secondary outcomes included preterm birth and low birth weight

**Results:**

Fifteen thousand four hundred fifty-nine women were available for final analysis, in which 1511 women resulted in twin delivery and 4788 women had singleton delivery. Female age over 35 was associated with reduced rates of twin pregnancy compared with female age at or less than 35 (9.5% vs 25.1%, aRR = 0.38 (0.27. 0.55)). Poor-type endometrium was associated with reduced rates of twin pregnancy (19.2% vs 27.5%, aRR = 0.75 (0.58. 0.96)). Two good-quality embryos for transfer was associated with significantly higher rates of twin pregnancy compared with one good-quality or none good-quality embryo (26% vs 12.8% vs 9.3%, aRR = 0.56 (0.45. 0.70), aRR = 0.44(0.26. 0.74)). Female age over 35 and none or one good-quality embryo for transfer were associated with reduced rate of low birth weight and preterm birth.

**Conclusion:**

Women with age over 35, poor-type endometrium, one good-quality embryo or none good-quality embryo were associated with reduced rate for twin pregnancy.

**Supplementary Information:**

The online version contains supplementary material available at 10.1186/s12884-022-04606-1.

## Background

In vitro fertilization (IVF) has allowed millions of infertile couples to get conception since the year 1978 [1]. IVF is more commonly used in recent years due to the increase of infertile couples [2–5]. However, IVF treatment is associated with the increased risk of several adverse obstetric and neonatal outcomes, including multiple pregnancy [6, 7], ovarian hyperstimulation syndrome (OHSS) [8], preterm birth [9, 10], low birthweight [11, 12], and small for gestational age [13–15].

Two or more embryo transfers have been used to increase the success rate of live birth in traditional IVF strategy [16, 17]. However, the risk of multiple pregnancy is also increased following the transfer of multiple embryos [18]. Multiple pregnancy is associated with increased risks of premature birth, placental related diseases (preeclampsia, gestational hypertension, placental abruption), cesarean section, and postpartum bleeding [19–24]. In addition, compared with singleton pregnancy, multiple pregnancy results in higher risks of several adverse neonatal outcomes, including low birth weight, fetal or neonatal death, and congenital malformations [25, 26]. Multiple pregnancy rate following IVF has been reduced over time due to the increased application of transfers with fewer and better-quality embryos [18, 27–29]. More recently, elective single embryo transfer has been increasingly popular. However, the decision towards elective single embryo transfer or double embryo transfer remains inconclusive up to now [30, 31], which needs to be further explored.

Previous studies demonstrated that the factors associated with twin pregnancy in women undergoing IVF include the increased height of women (> 1.74 cm), the younger age of women, a higher quality of transferred embryos, a high number of retrieved oocytes (> 8) [32–34]. However, due to the small sample size, the omission of some important covariables, and the heterogenous inclusion of participants, several potential risk factors for twin pregnancy may be neglected. Therefore, in this retrospective chort study, we aimed to explore the risk factors of twin pregnancy in women with double embryo transfer. We also investigated whether the risk factors of twin pregnancy were associated with the adverse outcomes, including preterm birth and low birth weight. We analyzed the data that include those with failed pregnancies after embryo transfer because we would like to highlight how these risk factors could be used as a tool for both pre-transfer and post-transfer counselling [35, 36]. This made our analyses more notable than previous studies which only looked at risk factors as a post-transfer counselling tool.

## Methods

This retrospective cohort study was approved by the Ethics Committee of Women’s Hospital of Zhejiang University. A total of 15,800 women who underwent their first double embryo transfer (DET) cycle from January 2010 to December 2018 in Women’s Hospital, Zhejiang University School of Medicine were enrolled in this study. Women with the following cycles were excluded: preimplantation genetic test (PGT); donor cycles; cyeles transferred with one; cleavage embryo and one blastocyst; cycles transferred with double blastocyst. Women who resulted in pregnancy was excluded if they had a triple pregnancy, monozygotic twins, fetal reduction, or loss of follow up. Gonadotropin-releasing hormone (GnRH) agonist or GnRH antagonist protocol was used for ovarian stimulation. Recombinant follicle stimulation hormone (FSH) (Gonafen, Pricon) and/or human menopausal gonadotrophin (Livzon, China) was commenced on day 2 or day 3 of the cycle at a dose of 75–225 IU, and the doses were adjusted according to the ovarian response (follicle count under ultrasound and/or serum E2 levels). When at least two leading follicles reached a size of around 18 mm, ovulation was induced by the administration of recombinant human chorionic gonadotropin (5000–6500 IU, Livzon, China); then, the oocyte was retrieved 36 to 38 h later. Fertilization was conducted by either conventional IVF or by an intracytoplasmic sperm injection (ICSI). Two embryos at the cleavage stage were transferred. The primary outcome was twin pregnancy defined as two live births after 22 weeks of gestation. Secondary outcomes included preterm birth (before 37 completed weeks of gestational age) and low birth weight (birth weight lower than 2500 g). The poor-type endometrium was defined as a multilayered endometrium consisting of prominent outer and midline hyperechogenic lines and inner hypoechogenic regions [37]. Cleavage embryos were graded according to the Istanbul consensus with 1 to 4 grade [38]. Grade I-II embryos were considered as good quality embryos, while Grade III-IV were considered as Poor-quality embryos. Thin endometrium was defined as endometrial thickness lower than 7 mm. Endometrial thickness at 7–14 mm was considered as the moderate endometrium and higher than 14 mm was considered as the thick emdometrium. High education was defined as a master degree or PhD degree. The number of oocyte retrieved at 0–5, 6–15, and > 16 were considered as poor ovarian response, moderate response, and high response, respectively.

Comparison between groups was performed using the independent sample t-test, Chi-square test (χ2), and non-parametric test as appropriate. Coefplot was used to visualize the risk factors for twin pregnancy, preterm birth, and low birth weight. A log-binomial regression model was used to calcualte the crude risk ratio (95% confidence interval) and adjusted risk ratio (95% confidence interval) of covaribles for the outcomes. The covariates included in the regression model included: female age (≤ 35 and > 35); male age (≤ 35 and > 35); female over weight (BMI < 25 and ≥ 25); male over weight: (BMI < 25 and ≥ 25); tubal factor; ovulatory dysfunction; male factor; endometriosis; endometrium type; sperm deviation; embryo quality; ICSI; smoking; endometrial thickness; the number of oocytes retrieve; Primary infertility; GnRH agonist and GnRH antagonist; education. All statistical procedures were run in Stata 15.1 (StataCorp LLC, Texas, USA). A *p*-value below 0.05 was considered as the statistical significance.

## Results

### Characteristics of enrolled patients

**Fig. 1:**
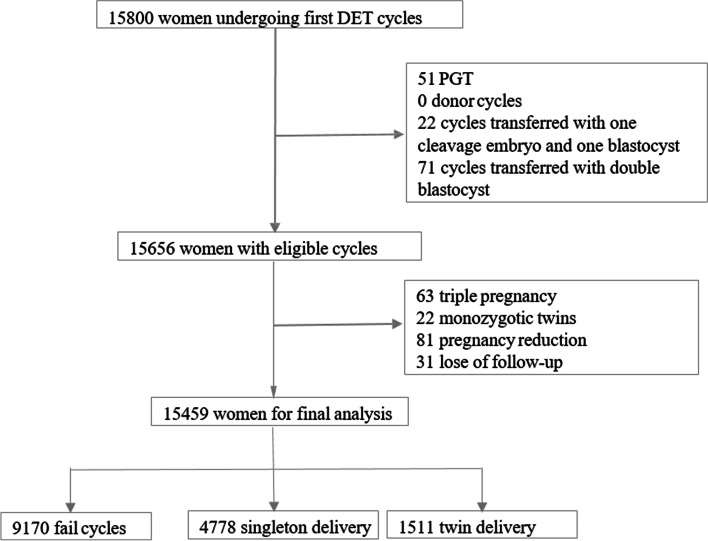
A flowchart of analysis

A total of 15,800 women who underwent their first DET cycle in the Women’s Hospital of Zhejiang University were enrolled. In these women, 51 were excluded because of PGT, none was excluded for donor cycles, 22 women were excluded due to the transfer of one cleavage embryo and one blastocyst, and 71 women were excluded due to double blastocyst transfer, 63 women were excluded because of triple pregnancy, 22 women were excluded with monozygotic twins, 81 women were excluded because the occurrence of pregnancr reduction and 31 women were excluded for lose of follow-up. At last, 15459 women were available for final analysis, in which 1511 women resulted in twin delivery, 4788 women had singleton delivery and 9170 women resulted in fail cycles (Fig. 1).

In women who underwent fresh embryo transfer cycles, twin pregnancy were associated with a reduced female age and male age, a higher rate of lower BMI, a higher number of oocytes, a thicker endometrium, a reduced years of infertity, a higher level of progesterone, a higher rate of endometriosis, GnRH agonist use, two good-quality embryos for transfer, high education, and high ovarian response (*P* < 0.001, *P* < 0.01, *P* < 0.01, *P* < 0.01, *P* < 0.01, *P* < 0.001, *P* < 0.01, *P* < 0.01, *P* < 0.01, *P* < 0.01, *P* < 0.01, *P* < 0.01) (Table 1). No statistically significant difference was found in male BMI, menarche, primary infertility, the level of FSH, LH, Estradiol and total testosterone, tubal factor, ovulatory dysfunction, male factor, sperm donor, fertilization methods, smoking among women resulted in twin pregnancy, single pregnancy and no pregnancy (Table 1).

**Table 1:** Characteristics of women undergoing fresh cycle

variables	Twins (*n* = 1511)	Singleton (*n* = 4778)	No delivery (*n* = 9170)	*P* value^a^
Female age y	29.4 ± 3.2	30.2 ± 3.7	30.7 ± 4.3	< 0.001
>35	41 (3%)	391 (8%)	1224 (13%)	
≤35	1470 (97%)	4387 (92%)	7946 (87%)	
Male age y	31.7 ± 4.3	32.1 ± 4.6	32.9 ± 5.3	< 0.001
>35	244 (16%)	991 (21%)	2424 (26%)	
≤35	1265 (84%)	3786 (79%)	6737 (74%)	
Female BMI kg/m^2^	21.8 ± 2.7	21.9 ± 2.8	22.1 ± 2.9	< 0.001
≥25	191 (13%)	661 (13.8%)	1450 ± (16%)	0.24
<25	1317 (87.3%)	4116 (86.2%)	7717 ± (84%)	
Male BMI kg/m^2^	23.7 ± 3.7	23.7 ± 3.6	23.7 ± 3.6	0.88
≥25	487 (33%)	1573 (33%)	2936 (33%)	
<25	992 (67%)	3135 (67%)	6038 (67%)	
Primary infertility	742 (49%)	2278 (48%)	4302 (47%)	0.25
FSH mIU/ml	7 (6–8)	7 (6–8)	7 (6–8)	0.44
LH mIU/ml	5 (3–6)	5 (3–6)	5 (3–6)	0.10
Estradiol pmol/l	113 (81–145)	111 (79–148)	114 (82–153)	0.008
Progesterone nmol/l	1.6 (1.1–2.3)	1.5 (1.1–2.1)	1.6 (1.1–2.2)	< 0.001
Total testosterone nmol/l	0.8 (0.5–1.1)	0.7 (0.5–1.1)	0.79 (0.5–1.1)	0.22
Reason for IVF				
Tubal factor	842 (56%)	2601 (54%)	4993 (54%)	0.64
Ovulatory dysfunction	85 (5.6%)	316 (6.6%)	643 (7.0%)	0.12
Male factor	447 (30%)	1586 (33%)	2960 (32%)	0.033
Endometriosis	188 (12.4%)	571 (12.0%)	866 (9.4%)	< 0.001
Down-regulation				< 0.001
GnRH agonist	1310 (86.7%)	3671 (76.8%)	7185 (78.4%)	
GnRH antagonist	182 (12%)	1019 (21.3%)	1807 (19.7%)	
Other	19 (1.3%)	88 (1.8%)	178 (1.9%)	
Sperm donor	27 (2.0%)	77 (2.1%)	108 (1.4%)	0.012
ICSI	392 (8.7%)	1392 (30.9%)	2723 (60.4%)	0.01
Poor-type endometrium	60 (4.8%)	253 (7.5%)	432 (6.0%)	0.001
Embryo quality				< 0.001
GG	1360 (92.2%)	3864 (82.2%)	7010 (78.1%)	
GP	97 (6.6%)	663 (14.1%)	1257 (14.0%)	
PP	18 (1.2%)	175 (3.7%)	712 (7.9%)	
High education	703 (46.6%)	2276 (47.7%)	3792 (41.4%)	< 0.001
Smoking				0.275
Yes	5 (0.3%)	27 (0.6%)	61 (0.7%)	
No	1505 (99.7%)	4747 (99.4%)	9102 (99.3%)	
Endometrial thickness mm	11.3 ± 2.4	11.1 ± 2.4	10.7 ± 2.4	< 0.001
Moderate	1288 (86%)	4068 (87%)	7938 (88%)	
Thin	18 (1%)	81 (2%)	271 (3%)	
Thick	184 (12%)	545 (12%)	824 (9%)	
Total number of oocytes	11.4 ± 4.8	10.9 ± 4.9	10.8 ± 5.0	< 0.001
Ovarian response				< 0.001
Moderate	1067 (71%)	3348 (70%)	6297 (69%)	
Poor	155 (10%)	639 (13%)	1349 (15%)	
High	289 (19%)	790 (17%)	1519 (17%)	

χ2, non-parametric test, or t test as appropriate. Data expressed with Mean ± SD or Median (IQR) or Number (percent) as appropriate. GG, two good-quality embryo; GP, one good-quality embryo and one poor-quality embryo; PP, two poor-quality embryo

Notes: Poor-type endometrium: A multilayered endometrium consisting of prominent outer and midline hyperechogenic lines and inner hypoechogenic regions. Good quality embryos: Grade I-II embryos; Poor-quality embryos: Grade III-IV embryos. Thin endometrium: Endometrial thickness lower than 7 mm; Moderate endometrium: Endometrial thickness at 7–14 mm; Thick endometrium: Endometrial thickness higher than 14 mm. High education: A master degree or PhD degree. Poor ovarian response: 0–5 oocytes retrieved; Moderate ovarian response: 6–15 oocytes retrieved; High ovarian response: > 16 oocytes retrieved

Sex hormones (FSH, LH, Progesterone, and Total testosterone) were measured on day 2–5 of menstrual cycle

**Fig. 2:**
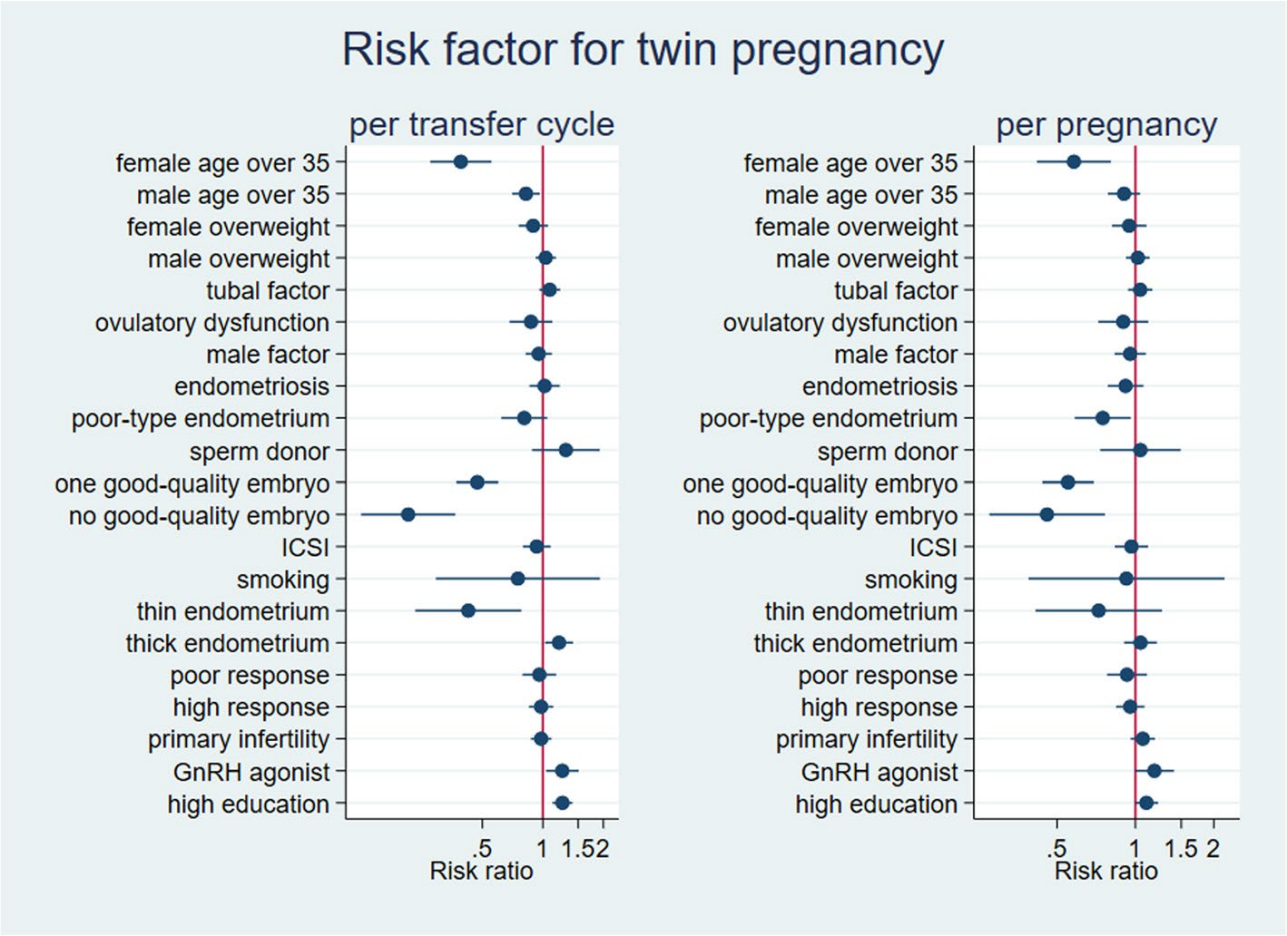
Coefplot visualizing the risk factors for twin pregnancy

In women who underwent fresh embryo transfer cycle, female age over 35 was associated with reduced rates of twin pregnancy compared with female age at or less than 35 (2% vs 11%, aRR = 0.38 (0.27. 0.55)). Women whose partner age was over 35 showed reduced rates of twin pregnancy compared with women whose partner age was at or less than 35 (7% vs 11%, aRR = 0.82 (0.70. 0.96)). Female who had two good-quality embryos was associated with higher rates of twin pregnancy compared with female who had one good-quality or none good-quality embryo (11.1% vs 4.8% vs 2.0%, aRR = 0.48 (0.38. 0.61), aRR = 0.21(0.12. 0.36)). Female with thin endometrium were associated with reduced rates of twin pregnancy (4.9%, aRR = 0.46 (0.26. 0.82)). Women with thick showed higher rates of twin pregnancy (11.8%, aRR = 1.21 (1.03, 1.43)). Women with GnRH agonist and high education were associated with a higher rate of twin pregnancy (10.8%, aRR = 1.22 (1.01, 1.46), 10.4%, aRR = 1.23 (1.09, 1.38). Female overweight, male overweight, the reasons for IVF including tuabal factor, ovaulatory dysfunction, male factor and endometriosis, endometrium type, sperm donor, fertilization method, smoking, poor or high ovarian response and primary infertility were not associated with twin pregnancy rates (Fig. 2).

In women who resulted in pregnancies, female age over 35 was associated with reduced rates of twin pregnancy compared with female age at or less than 35 (9.5% vs 25.1%, aRR = 0.38 (0.27. 0.55)). Women with poor-type endometrium was associated with reduced rates of twin pregnancy (19.2% vs 27.5%, aRR = 0.75 (0.58. 0.96)). Female who had two good-quality embryos for transfer showed significantly higher rates of twin pregnancy compared with female who had one good-quality or none good-quality embryo (26% vs 12.8% vs 9.3%, aRR = 0.56 (0.45. 0.70), aRR = 0.44(0.26. 0.74)). We found no significance in female and male overweight, the reasons for IVF (tubal factor, ovulatory dysfunction, male factor, endometriosis), poor-type endometrium, fertilization methods, smoking, with the proportion of thin or thick endometrium, ovarian response, primary infertility, GnRH agonist, and high education (Fig. 2).

Women with age over 35 and women with none or one good-quality embryo for transfer were associated with reduced rate of low birth weight (per transfer cycle and per pregnancy, Supplemental Figure 1). However, women with poor-type endometrium, high education, and thick endometrium were associated with increased rate of fetal low birth weight (per transfer cycle, Supplemental Figure 1).

Women with age over 35 and women with none or one good-quality embryo for transfer were associated with reduced rates of preterm birth (per transfer cycle and per pregnancy, Supplemental Figure 2). In women underwent per transfer cycle, women with high education and women with thick endometrium were associated with increased rate of pretern birth (per transfer cycle Supplemental Figure 2).

## Discussion

In this study, we showed that women with age over 35, one good-quality embryo and none good-quality embryo were associated with reduced rate for twin pregnancy (per cycle and per pregnancy). There was a trend towards an increased rate of twin pregnancy in women with high education and women with GnRH agonist.

Previous studies demonstrated that the good-quality embryos and a high number of retrieved oocytes increase the chance of dizygotic twinning after IVF with DET [32, 39–42]. Consistent with previous studies, our data demonstrated that women transferred with two good-quality embryos showed higher rates of twin pregnancy compared with women transferred with one or none good-quality embryo. However, our study demonstrated that women with high ovarian response were not associated with increased rates of twin pregnancy. Due to the inadvanced embryo frozen technology in the past decade, the strategy of transferring multiple embryos is often preferred, which leads to increased rates of twin pregnancy. Our research included women who underwent fresh embryo transfer, and if the number of oocytes retrieved was greater than 20, we generally chose to freeze all embryos instead of fresh transfer. Table 1 showed that the proportion of women with high ovarian response is relatively low and the average number of oocytes is around 11. On the other hand, previous studies have often decided to transfer two or more embryos in women with a large number of oocytes, which leads to an increase rate of twin pregnancy [42, 43]. These data may explain the difference between our study and previous studies regarding to the association of high response with twin pregnancy rate. Furthermore, our data also demonstrated that women with good-type endometrium and age under 35 were associated with a higher rate of twin pregnancy, which have not been determined in previous studies. Therefore, these women need to be cautious when choosing double embryo transfer because the rates of twin pregnancy tend to increase in these women.

At the same time, we also explored the risk factors of premature birth and low birth weight infants. Our datas showed that women with age under 35 and women transferred with two good-quality embryos were associated with a higher rate of twin pregnancy as well as preterm birth and low birth weight. In addition, previous studies have shown that twin pregnancy may lead to premature birth and low birth weight infants [44, 45]. Overall, these data strongly suggested the adverse outcomes in women with age under 35 and women transferred with two good-quality embryos. Thus, an elective single embryo transfer should be considered in women with age under 35 and with good-quality embryos. Additionally, there is a trend towards an increased risk of twin pregnancy, perterm birth, and low birth weight in women with high education and women with GnRH agonist use. One study explored the association between educational attainment and twin pregnancies, and the study found no significant association [32]. Our study found there is a trend towards an increased risk of twin pregnancy in women with high education, the result could be random and should be further confirmed by future studies with large sample size. An elective single embryo transfer should also be preferred in these women.

Our study has several limitaions. Duo to the retrospective nature, some potential factors that may be associated with the studied outcomes were not included. Additionally, the obstetric outcomes were not available to analyse in our dataset.

## Conclusions

In the present study, we showed that women with age over 35, one good-quality embryo and none good-quality embryo were associated with reduced rate of twin pregnancy (per cycle and per pregnancy). There is a trend towards an increased rate of twin pregnancy in women with high education and women with GnRH agonist.

## Supplementary Information

**Additional file 1.**

**Additional file 2.**

## Data Availability

All generated data are incorporated into the article and its online supplementary material. The original data of individual participants underlying this article will be shared on reasonable request to the corresponding author.

## Abbreviations

IVF: In vitro fertilization
GnRH: Gonadotropin-releasing hormone
OHSS: Ovarian hyperstimulation syndrome
DET: Double embryo transfer
PGT: Preimplantation genetic test
FSH: Follicle stimulation hormone
ICSI: Intracytoplasmic sperm injection
