# Supplementary figures and images for: Risk factors for twin pregnancy in women undergoing double cleavage embryo transfer
Source: BMC Pregnancy Childbirth. 2022 Mar 29;22:264. doi: 10.1186/s12884-022-04606-1 (PMC8966328; doi:10.1186/s12884-022-04606-1)

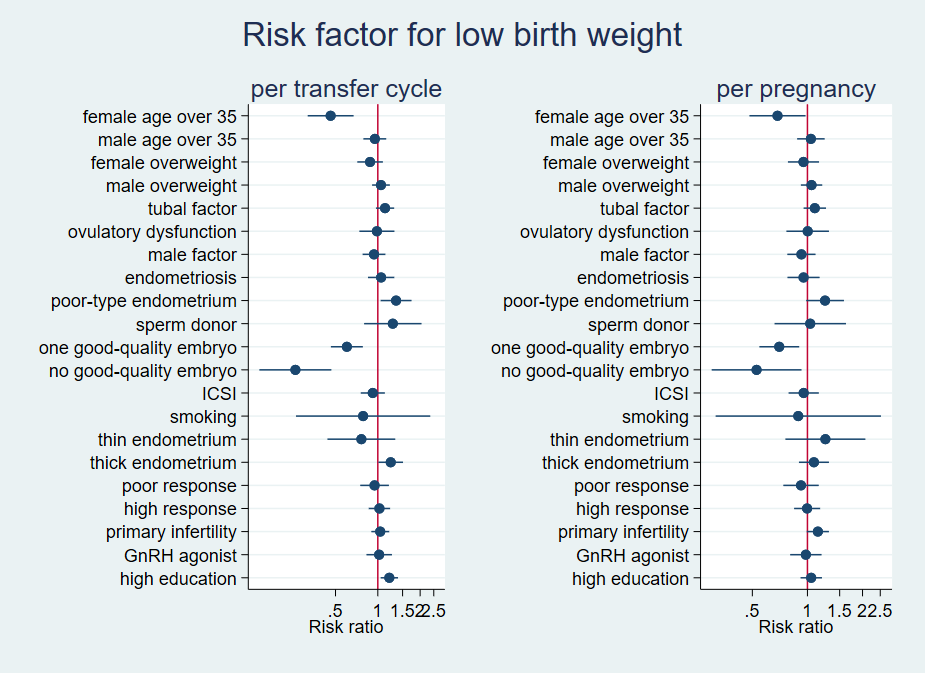

Supplement: Supplementary file 1 — Additional file 1. [file 12884_2022_4606_MOESM1_ESM.tif]

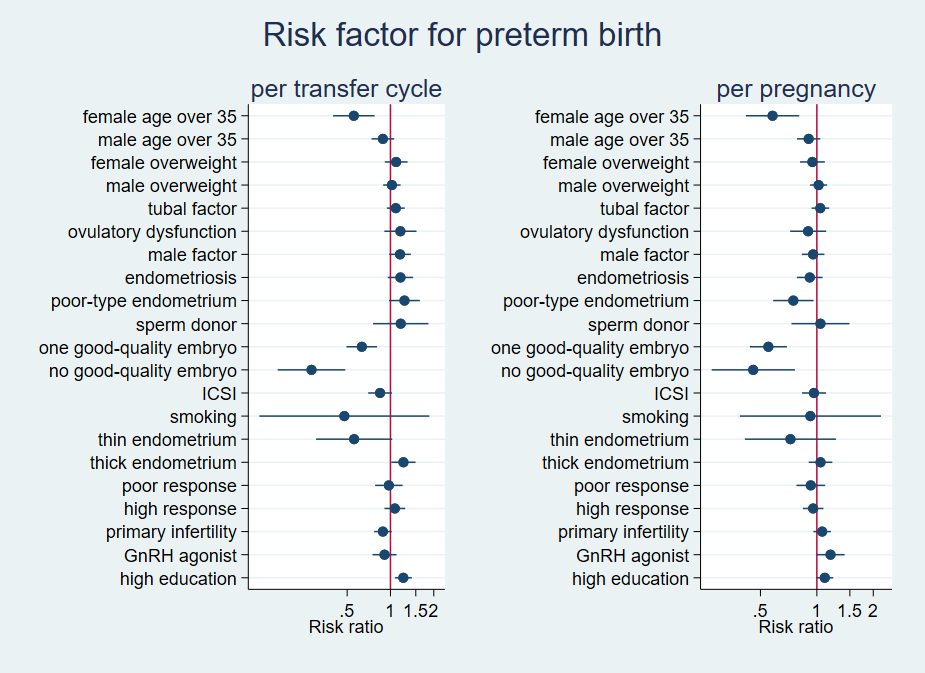

Supplement: Supplementary file 2 — Additional file 2. [file 12884_2022_4606_MOESM2_ESM.tif]
